# IP-10 for the diagnosis of tuberculosis in children: Protocol for a systematic review and meta-analysis

**DOI:** 10.1097/MD.0000000000015977

**Published:** 2019-06-07

**Authors:** Weijun Zhang

**Affiliations:** Department of Clinical Laboratory Center, the Second Hospital of Lanzhou University, Lanzhou, China.

**Keywords:** children, diagnosis, IP-10, meta-analysis, systematic review, tuberculosis

## Abstract

**Background:**

Tuberculosis (TB) is a highly contagious and chronic disease. The microbiological examination to confirm children TB disease are limited due to paucibacillary Mycobacterium, specimens and detecting facilities. Considering these limitations in diagnosing children TB, new and reliable methods that detect children TB should be developed. Recently, Interferon gamma-induced protein 10 (IP-10) has been identified as a sensitive parameter in detecting children TB. The present study aims to synthesis and analysis the diagnostic value of IP-10 for children TB.

**Methods:**

We will search PubMed, Embase, the Cochrane Library, Web of Science, Chinese National Knowledge Infrastructure, and Chinese Biological Medical Databases. We will search relevant citations up to May 2019. The quality of individual study will be assessed using the Quality Assessment of Diagnostic Accuracy Studies tool-2 (QUADAS-2). Stata 14.0 software will be used to calculate the pooled sensitivity, pooled specificity, pooled positive likelihood ratio (PLR), pooled negative likelihood ratio (NLR), pooled diagnostic odds ratio (DOR), pre-test probability, post-test probability and the hierarchical summary receiver operating characteristic (HSROC) curve.

**Results:**

The results of this study will be published in a peer-reviewed journal.

**Discussion:**

The evidence will indicate that IP-10 test is an alternative immunological test in detecting children TB. This is a protocol of systematic review and meta-analysis, so the ethical approval and patient consent are not required.

**Protocol registration number:**

CRD42019129743.

## 1 Introduction

### 1.1 Target condition

Tuberculosis (TB) is a highly contagious and chronic disease.^[1]^ Although the management of TB has been quickly improved, it is still a major health and economic burden.^[2]^ World Health Organization (WHO) estimates that there will be approximately 10 million newly diagnosed TB individuals in the world and more than 1.3 million died from the disease in 2017.^[3]^ Children TB has contributed a lot to TB morbidity and mortality.^[4]^ More than 0.5 million children develop TB and 0.07 million patients die each year.^[5]^ Additionally, the young TB-infected children, especially 2 years old, are at the peak of developing active TB.^[6]^ To date, in order to eliminate children TB, a major goal is to correctly discriminate it.

### 1.2 Clinical practice and the importance to perform the review

Diagnostic children TB correctly is difficult due to the wide spectrum of clinical presentation and several disadvantages of detect methods.^[7]^ In clinical practice, cough, the most common clinical symptoms of children TB, is non-specific.^[8]^ Chest X-ray, one of routine children TB diagnostic methods, should be used with other methods. The results of microbiological examination, nucleic acid amplification and acid-fast bacillus stains confirmation children TB disease are limited because of paucibacillary Mycobacterium, specimens and detecting facilities.^[9,10]^ Immunological tests are also crucial for detecting TB among children. The tuberculin skin tests (TSTs), is low specificity and depends on operators.^[11]^ Interferon-gamma release assays (IGRAs) detect many indeterminate results in TB children.^[12,13]^ Therefore, considering these limitations in diagnosis of children TB, new and reliable methods that detect children TB should be developed.

Interferon gamma-induced protein 10 (IP-10), a 7.2-kilodalton chemokine, could express 100-fold higher levels than IFN-gamma after TB infection.^[9,15]^ The individuals’ age, gender and TB presentation do not affect the level of IP-10.^[14–16]^ In recent years, many studies have reported that IP-10 has been identified as a sensitive parameter in detecting children TB.^[9,17,18]^

### 1.3 Objectives

The diagnostic value of IP-10 for children TB has been reported in many studies; however, the results are variable. Therefore, our study aims to synthesis and analysis the diagnostic value of IP-10 for children TB.

## 2 Methods

This protocol will be reported according to preferred reporting items for systematic review and meta-analysis protocols (PRISMA-P).^[19]^ This study will be performed and reported following the Preferred Reporting Items for Systematic Reviews and Meta-Analyses diagnostic test accuracy criteria.^[20]^ Besides, the protocol has been registered on the international prospective register of systematic review (PROSPERO) (CRD42019129743). Because this study is based on published trials, analyses of data can be done without patient consent and ethical approval.

### 2.1 Eligibility criteria

#### 2.1.1 Type of studies

We will include case-control studies and cohort studies, exclude animal experiments, only abstracts without full texts, duplications of the same trials, letters and reviews.

#### 2.1.2 Participants

The age of patients is less than 18 years old, including neonates, infants, children and adolescents. Both of patients with TB and suspected TB can be included. The sample size of eligible studies should be more than 5. There will be no restrictions on publication date and publication language.

#### 2.1.3 Index test

We will only include studies of IP-10 for the detection of children TB as the index test. The specimen sources will be sputum, gastric aspirate, blood and pleural effusion.

#### 2.1.4 Reference standards

The first reference standard is culture-positive and/or clinical and radiological tests, which is called “confirmed children TB”. Second reference standard is based on clinical presentation and radiological confirmation if the culture is negative, which is called “clinical children TB”.

#### 2.1.5 Outcomes

The outcomes of interest include sensitivity and specificity of IP-10 for detecting children TB.

### 2.2 Literature search

We will search PubMed, Embase, the Cochrane Library, Web of Science, CNKI (Chinese National Knowledge Infrastructure), and CBM (Chinese Biological Medical Database) databases from their inception up to May 2019. The search terms include “tuberculosis”, “mycobacterium tuberculosis”, “TB”, “children”, “neonate”, “infant”, “adolescent”, “Interferon gamma-induced protein 10” and “IP-10”. The references of relevant reviews will be manually tracked to identify additional studies. The PubMed search strategies as follows,

#1 “Tuberculosis” [Mesh]
#2 tuberculosis [Title/Abstract] OR mycobacterium tuberculosis [Title/Abstract] OR TB [Title/Abstract] OR tuberculoses [Title/Abstract] OR mycobacterium tuberculosis Infection [Title/Abstract] OR tuberculosis infection [Title/Abstract]
#3 #1 OR #2
#4 “Chemokine CXCL10” [Mesh]
#5 Cytokine IP 10 Protein [Title/Abstract] OR IP-10 [Title/Abstract] OR interferon gamma-induced protein 10 [Title/Abstract] OR interferon-inducible protein 10 [Title/Abstract] OR CXCL10 [Title/Abstract] OR Chemokine CXCL10 [Title/Abstract]
#6 #4 OR #5
#7 #3 AND #6 Filters: Child: birth-18 years.

### 2.3 Literature selection

**Figure 1:**
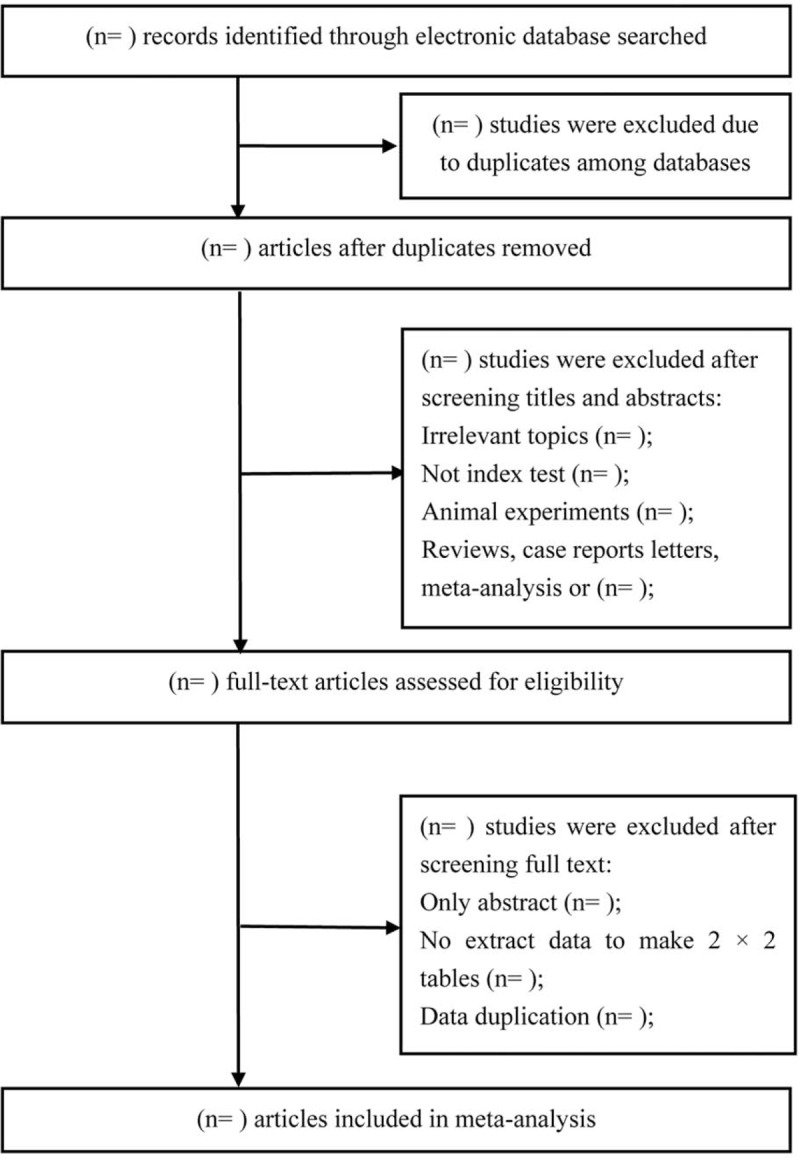
Study selection.

Initial search records will be imported into the EndNote version 8. After the duplicated records are deleted, the titles and abstracts of records will be reviewed independently to identify potential studies according to eligibility criteria. Then, the full-texts of all potentially relevant studies will be downloaded to confirm eligible studies. Any conflict will be resolved by discussion. Study selection will be shown in Figure 1.

### 2.4 Data extraction

Data extraction will be independently performed by 2 reviewers. Any disagreements will be resolved by discussing together. The characteristic data include: author, published date, study location, TB high burden (yes/no), study design type, number of participants, TB site (pulmonary TB and/or extra-pulmonary TB), age of participants, sex, index test (measurement method, condition, species and cut-off value), BCG vaccinates, co-infection.

The primary data will include true positive (individuals with positive IP-10 who have been proven children TB), false positive (individuals with positive IP-10 who do not have been proven children TB), false negative (individuals with negative IP-10 who have been proven children TB), true negative (individuals with negative IP-10 who do not have been proven children TB), sensitivity and specificity of IP-10 for diagnosing children TB.

### 2.5 Quality assessment

Two reviewers will independently assess the quality of individual study using Quality Assessment of Diagnostic Accuracy Studies tool-2 (QUADAS-2).^[21]^ In terms of patient selection, bias will exist when the selection criteria are not clearly described. In terms of index test, bias will exist when no sufficient details of IP-10 test describe in the eligible studies. In terms of reference standard, bias will exist when there is no enough time between the reference test and IP-10 test for detecting children TB. In terms of flow and timing, bias will exist when the test results reported are not interpretable. For unresolved disagreements, a third reviewer will be consulted. RevMan 5.3 software will be used to summary the quality of individual study.

### 2.6 Statistical analysis

Stata 14.0 software will be used to perform the statistical analysis and draw funnel plots. Heterogeneity is obtained by Cochrane Q test and I^2^ statistic.^[22]^ I^2^ can be calculated from the formula of I^2^ = 100% × (Q – df) / Q. If I^2^ is < 50%, a fixed effect model is used for pooling the data; whereas, if I^2^ is >50%, then there is more heterogeneity among studies, and a bivariate random effects model could be utilized to analyze the data. We will conduct the pooled sensitivity, specificity, positive likelihood ratio (PLR), negative likelihood ratio (NLR), diagnostic odds ratio (DOR), pre-test probability, post-test probability and draw the hierarchical summary receiver operating characteristic (HSROC) curve to evaluate the diagnostic accuracy of IP-10 for children TB.^[23]^

Additionally, we will conduct the Galbraith plot analysis, sensitivity analysis and subgroup analyses to explore the potential sources of heterogeneity, including TB high burden (yes/no), HIV infected (yes/no), the method of index test, the condition of index test and cut-off value. The Deek funnel plot will be used to assess the publication bias when more than 10 studies are included.^[24,25]^

## Author contributions

ZWJ planned and designed the research; ZWJ tested the feasibility of the study; ZWJ wrote the manuscript and approved the final version of the manuscript.

**Conceptualization:** Weijun Zhang.

**Investigation:** Weijun Zhang.

**Methodology:** Weijun Zhang.

**Project administration:** Weijun Zhang.

**Software:** Weijun Zhang.

**Writing – original draft:** Weijun Zhang.

**Writing – review & editing:** Weijun Zhang.
